# Gonad pathology, sex hormone modulation and vitellogenin expression in *Chrysichthys nigrodigitatus* from Lagos and Epe lagoons within the southern-lagoon system, Nigeria

**DOI:** 10.3389/ftox.2024.1336916

**Published:** 2024-02-06

**Authors:** Olusola A. Akangbe, Azubuike V. Chukwuka, Maurice E. Imiuwa, Aina O. Adeogun

**Affiliations:** ^1^ Department of Zoology, University of Ibadan, Ibadan, Oyo, Nigeria; ^2^ National Environmental Standards and Regulations Enforcement Agency (NESREA), Wupa, Nigeria; ^3^ Department of Animal and Environmental Biology, University of Benin, Benin, Nigeria

**Keywords:** environmental estrogens, *Chrysichthys nigrodigitatus*, vitellogenin protein, gonadal alterations, intersex, endocrine disruption

## Abstract

**Introduction:** Estrogenic chemicals in aquatic environments impact fish reproductive health, with vitellogenin protein levels serving as a crucial biomarker for xenoestrogen exposure. Limited knowledge exists on estrogenic effects in tropical environments, prompting an investigation into the influence of environmental estrogens on *Chrysichthys nigrodigitatus* in Lagos and Epe lagoons.

**Methods:** A total of 195 fish samples underwent analysis for vitellogenin protein, sex hormones (testosterone and 17 β-estradiol), and gonad pathology in effluent-receiving areas of the specified lagoons.

**Results:** Gonadal alterations were observed in male and female fish, including empty seminiferous tubules and distorted ovaries. Intersex occurred in 3.81% of Lagos and 3.33% of Epe. Testosterone levels were generally higher in females and males from both lagoons, while E2 levels were higher in females from both lagoons, with Lagos showing higher levels than Epe. Vtg levels were higher in males than females in Lagos samples but showed no significant difference in Epe samples.

**Discussion:** Contaminant analysis revealed similar trends in metals (Hg, As, Cr) and phthalates (DEHP, DBP, DEP) in both sexes in the Epe population. Multivariate depictions from the PCA showed sex-specific patterns of metal uptake (Cd) in male fishes at the Lagos Lagoon. The positive association between higher pH loadings and metal and DBP levels in sediment at the Lagos lagoon suggests the influence of higher alkalinity in lower bioavailability of contaminants.

**Conclusion:** Endocrine disrupting effects were observed in male and female *Chrysichthys nigrodigitatus* in Lagos and Epe lagoons populations, with notable differences in hormone and contaminant concentrations between the two lagoon systems. Identification of specific contaminants and their spatial and temporal trends can inform targeted management and remediation efforts to protect and restore these valuable aquatic ecosystems.

## Introduction

The pollution of aquatic ecosystems and implications for local and regional fisheries are issues of important societal and scientific concerns because this has direct bearing on the sustainability of ecosystem services (Häder et al., 2020). Some of the most significant and far-reaching deleterious effects have been attributed to the introduction of chemicals with endocrine disrupting effects into waterways through anthropogenic activities and the resultant exposure of fauna populations in affected aquatic systems (Adeogun et al., 2016b). Adverse impacts have been noted at minimal concentrations of endocrine-disrupting chemicals (EDCs) in the environment, underscoring the potential risks associated with exposure (Walf et al., 2011; Windsor et al., 2018). While reports of endocrine-disrupting effects in fish have demonstrated marked hormonal imbalance by mimicking endogenous estrogen (Rehberger et al., 2020), effects in gonochoristic fish (separate sexes) has also emerged as a concern (Muller et al., 2020). The spectrum of EDC-related effects includes the disrupted process of sexual differentiation and altered gonadal development resulting in temporary or permanent damages to reproductive systems (Naderi et al., 2014; Luzio et al., 2016) including the development/incidence of intersex individuals within exposed populations (Barnhoorn et al., 2010). Other markers of endocrine disruption reported within fish species include modulated production of sex hormones and vitellogenin (Adeogun et al., 2016b; Ibor et al., 2016).

Several studies have indicated a correlation between hormone dynamics, stages of gonadal development, and fish maturation events (Maitra et al., 2013; Díaz and Piferrer, 2017; Amenyogbe et al., 2020). EDCs including metals and phthalates entering aquatic habitats from diffuse sources interfere with hormonal systems and impact the reproductive health of local populations (Gonsioroski et al., 2020). The expression of liver-synthesized vitellogenin (a female-specific precursor protein) in male and juvenile fish is considered an important physiological indicator of endocrine disruptive effects (Hara et al., 2016). As such, the expression of this protein is considered a classical indicator of endocrine disruption in captive and wild fishes and has been linked with the presence of EDCs in several studies (Adeogun et al., 2016a; Ibor et al., 2016). Several reports have indicated that intersex, inhibited gonadal development and aberrations, alteration of sex steroid hormone and vitellogenin levels are related to exposure to endocrine-disrupting chemicals (Adeogun et al., 2019; Delbes et al., 2022).

Tropical lagoons including those off the Gulf of Guinea like Lagos and Epe lagoons are characterized by a wide biologically diverse and highly productive brackish stretch of lagoon systems surrounding the island of Lagos and impacted by unabated pollution incidence which has negative implications for survival of local fisheries (Olakolu and Chukwuka, 2014; Adeogun et al., 2015a). The presence of EDCs originating from various industrial and domestic activities in the southern-lagoon system of Nigeria has been reported (Adeyi, 2020; Jerome et al., 2020). The unabated and indiscriminate discharge of untreated effluents from these adjacent land-based sources into the Lagos Lagoon has also been documented (Adeogun et al., 2015b; Jerome et al., 2017a). The Silver catfish, *Chrysichthys nigrodigitatus* is one of the most landed fisheries from the southern-lagoon system of Nigeria (Abidemi-Iromini et al., 2019) and a species of interest following its habitat range within the Lagos and Epe lagoons and benthopelagic preference that ensures significant risks of contaminant uptake via considerable contact with sediment repositories (Olarinmoye et al., 2011; Kanu and Idowu, 2017). However, knowledge gaps on pollution risks for this catfish based on gross gonadal examination and implications for other similar lagoon species is considerably large. This study seeks to provide a comparative report of the relationship between the presence of EDCs and markers of endocrine disruptions in *Chrysischthys nigrodigitatus* populations exposed to phthalates and metals in Lagos and Epe lagoons. While seeking to uncover potential endocrine disparities and deepen our understanding of environmental influences on the reproductive health of *Chrysichthys nigrodigitatus* in the Southern-Lagoon system, we specifically hypothesize that *Chrysichthys nigrodigitatus* in Lagos and Epe Lagoons may exhibit sex-specific differences in hormone modulation, driven by environmental factors.

## Materials and methods

### Site description

**FIGURE 1:**
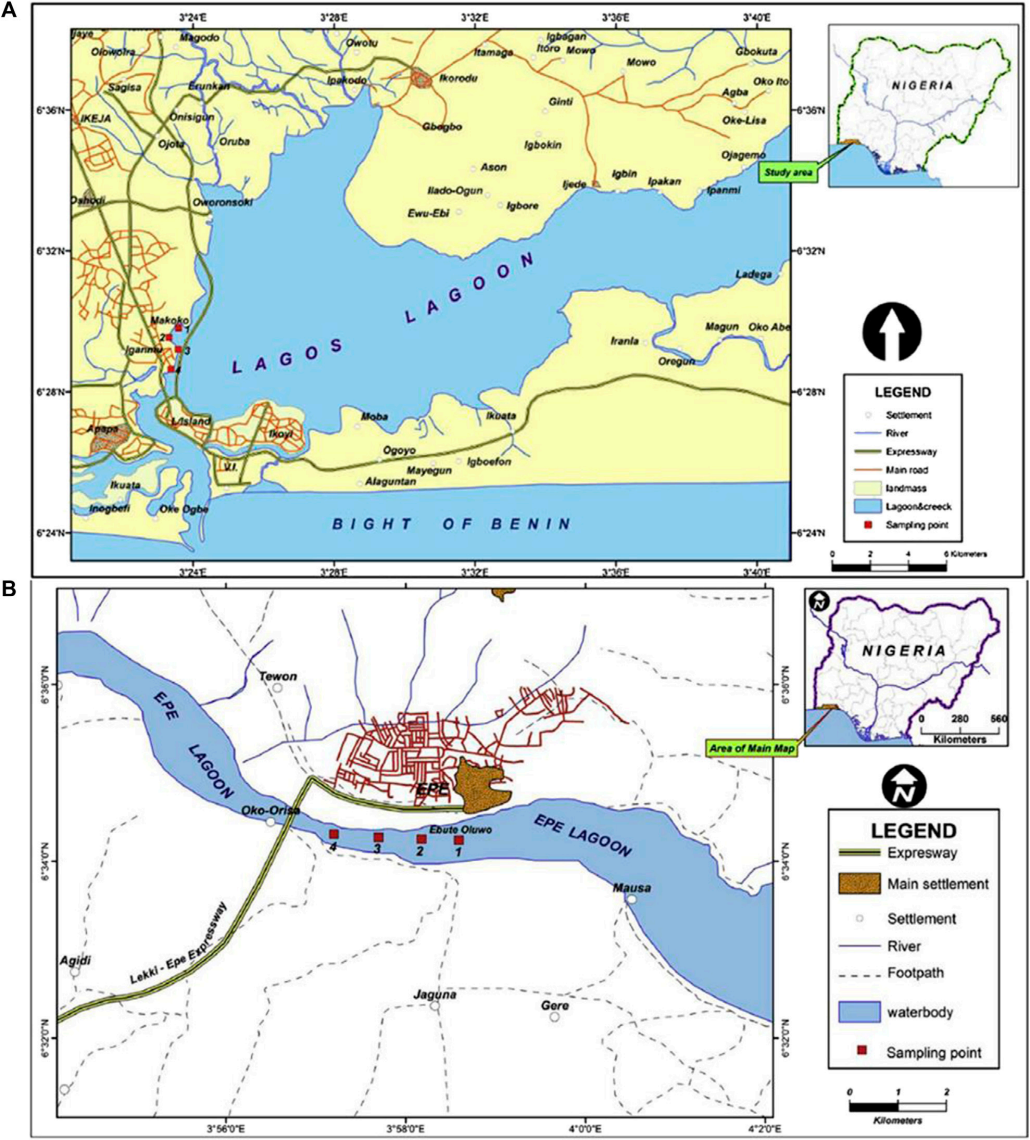
Map showing sampling sites. **(A)** Lagos lagoon. **(B)** Epe lagoon.

Lagos lagoon is the largest of four lagoon systems in the Gulf of Guinea (Webb, 1958) and stretches for about 250 km from Cotonou in the Republic of Benin to the western edge of the Niger Delta. The lagoon includes the forest belt and receives several important large rivers such as Yewa, Ogun, Ona, and Osun rivers, draining more than 103,626 km^2^ of the country and empties into the Atlantic Ocean (Figure 1A). The Lagos opening is the largest and forms an extensive harbor, which serve as the major outlet of fresh water from the lagoon system during the rainy season. The central body of the lagoon is located between longitude 3° 23′0° and 3° 40′0 E and latitude 6° 22′0° and 6° 38′0 N. The brackish region is a significant area of concern for the transportation of pollutants from both the hinterland and the immediate shores of the lagoon (Ajao and Fagade (1990). Due to the fact that the lagoon watershed encompasses both residential and industrial areas, it is frequently used as a dumping site for untreated anthropogenic effluent. (Jerome et al., 2017b).

The surface area of the Epe lagoon spans approximately 247 km^2^, with a maximum depth of 6.4 m and shallow regions accounting for most of the lagoon and measures less than 3.0 m deep. It is situated between latitudes 6° 25′ and 6° 37′N, and longitudes 4° 00′ and 4° 15′E. The lagoon receives River Osun that drains a number of cities and agricultural lands (Figure 1B). The study area is bordered on the west by a number of cultivated lands and receives wood wastes from local wood processing industries located at the bank of the lagoon. The lagoon is used for transportation of timber logs (possible source of wood particles and leachates) from the villages to the city of Lagos and is the second largest contributor to the viable commercial artisanal fisheries of the southern lagoon complex. The lagoon houses a major jetty at Epe, where different forms of anthropogenic wastes within and around the jetty are indiscriminately deposited (Edokpayi et al., 2010).

### Sample collection

Fish and sediment samples were collected from the Makoko site of Lagos Lagoon where anthropogenic activities such as fishing and transportation are common as well as domestic waste discharge from residencies on this axis of the Lagoon. A long stretch of large sawmills and wood processing industries that dump sawdust into the lagoon at this site. Similarly, fish was collected at the Oluwo landing jetty of Epe Lagoon where several commercial activities and anthropogenic wastes can be found. A total of 195 samples were collected comprising 105 samples from Lagos Lagoon and 90 samples from Epe Lagoon with the aid of artisanal fishermen using a combination of gill and cast nets (mesh size 50–55 mm). This was done between 6–11 a.m. once a month from Nov. 2020 to May. 2021. Sediment samples were also collected in duplicates on-site using a van Veen grab at points close to where fish were sampled, wrapped with foil paper and kept at −20°C before extraction for contaminant analysis (Frias et al., 2018).

Blood samples were quickly collected on site from the caudal vein of *C. nigrodigitatus* using a sterile 2 mL syringe and transferred into a 5 mL Heparin vacutainer (Denslow et al., 1999). Serum was separated from the blood by placing the vacutainer in a standing position and the supernatant was placed in 1.5 mL cryogenic polypropylene Eppendorf tubes, preserved in ice, and transferred to the laboratory pending further analysis. Fish samples were tagged, preserved on ice, and transferred to the laboratory for further analysis.

### Biometric analyses

#### Morphometric measurements

Morphometric parameters measured with an Ohaus digital meter (Mettler Instruments) and an Absolute Digital Caliper (Tresna Instruments) were standard length (SL), total length (TL), and Wet weight (W). Condition index was also calculated using the Fulton’s formula.

Condition Factor (k) = 100 W⁄L^3^ where W is wet weight (g) and L is total length (cm).

#### Gonad evaluation

Gonads were harvested, observed macroscopically, and classed into stages of development. As a gonochoristic species with distinct male and female individuals, sex identification relied on external and internal morphological characteristics (Rodriguez et al., 1997; Edem et al., 2021). Genetic analyses were omitted from the sex determination process. Acknowledging the potential influence of endocrine-disrupting chemicals (EDCs) on sex ratios and gonad morphology in species with genetic sex determination (Scholz and Klüver, 2009), this was recognized as a study limitation.

For histological examination, excised gonads were placed in Bouin’s fluid for 72 h to enable tissue hardening (Culling, 1974). Gonads were then transferred to 10% phosphate-buffered formalin for preservation, dehydrated in a graded series of ethanol dilutions, and embedded in paraffin wax (Barnhoorn et al., 2010). Section of 5 µm was cut and stained with hematoxylin and eosin (H&E) and examined (Liu et al., 2018). Over the 7-month study duration, six individuals each (male and female per site) were sampled each month per site, and three sections were obtained for each sampled individual.

The gonadosomatic index (GSI), was calculated according to the equation: gonad weight/(body weight – gonad weight) × 100.

#### Plasma sex hormones and vitellogenin quantification

Sex hormones (17β-estradiol and testosterone) were measured in fish serum using ELISA kits from Randox Laboratories. For estradiol quantification, 15 µL of plasma samples and standard solution were added to a pre-coated microliter plate, incubated for 60 min, and washed before adding TMB. The color reaction was stopped, and the intensity of the color was measured at 450 nm using a Robonik 11–2,000 ELISA plate reader after 15 min. For testosterone quantification, 25 µL plasma sample were pipetted into pre-coated microliter plate, and HRP substrate was added 4 times before incubating at 37°C for 60 min. After washing the plate, TMB was added and further incubated for another 60 min at the same temperature. The color intensity (OD) was measured for 20 min after the addition of 1N HCl, at 450 nm using a Robonik 11–2000 ELISA plate reader.

In this study, a highly sensitive Fish Vitellogenin ELISA Kit (Bioassay Technology Laboratory) was procured to measure the serum levels of VTG in fish blood. The kit had a sensitivity of 0.55 μg/mL, allowing for precise measurements of VTG levels and the standards used in the study were prepared following the manufacturer’s protocol. To ensure the reliability and validity of the VTG measurements obtained in this study, a standard curve was generated using the standards, which fell within the recommended range advised by the manufacturer (Supplementary Material SI).

#### Quantification of chemicals in fish tissue and sediment

The levels of five heavy metals/metalloids: Chromium, Cadmium, Lead, Arsenic, and Mercury, in sediment and fish muscle samples were quantified using Atomic Absorption Spectrophotometer (AAS). Three phthalic esters (DEHP, DEP, DBP) that were previously identified and quantified in sediment were included in chemical analysis (Adeogun et al., 2015b).

For the analysis of fish muscle samples, 2 g of flesh (wet weight) was weighed into an open beaker, and 10 mL of 1:1 Nitric acid – Hydrogen peroxide was added. The beaker was covered with a watch glass and left for 1 h. Subsequently, the beaker was placed in a water bath and heated gradually to 160°C, and the content boiled for about 2 h (Aderinola, et al., 2012). The digested sample was allowed to cool and made up to 25 mL with de-ionized water for AAS analysis.

Sediment samples were air-dried, pulverized and sieved with a 2 mm sieve. To this, 9 mL of Nitric acid (65%) and 3 mL of HCL (37%) in a ratio of 3:1 was added to 5 g of sediment sample, and the mixture was digested for 1 h at 160°C (Uddin et al. (2016) After cooling, the solution was filtered into a volumetric flask, and deionized water was added to make the total volume up to 100 mL. The resulting sample solution was then transferred to sample bottles for analysis of metals (Hg, Cd, Cr, Pb, and As) using an Atomic Absorption Spectrophotometer (AAS). The recovery rates for the analyzed metals were within acceptable ranges: Mercury (Hg) demonstrated a recovery rate between 95% and 105%, Cadmium (Cd) ranged from 90% to 110%, Chromium (Cr) showed a recovery rate between 95% and 105%, Lead (Pb) fell within the range of 90%–110%, and Arsenic (As) exhibited a recovery rate between 95% and 105%. These recovery rates indicate a high level of accuracy in the analytical procedures employed for metal analysis in the sediment samples.

#### Phthalate sample preparation, extraction and analysis

Muscle samples weighing 10 g were collected and homogenized into a paste-like consistency using a glass mortar and pestle. The resulting mixture was then dried with anhydrous sodium sulfate, following the USEPA (2012) protocol. For the water samples, 200 mL was collected and spiked with butyl benzoate, and 6 g of sodium chloride was added to prevent persistent emulsion. Three portions of 25 mL dichloromethane (DCM) were used for extraction. To remove free fatty acid (FFA) interferences, further extraction with sodium carbonate was carried out. The organic extracts were then dried with anhydrous sodium sulfate, as described by Ogunfowokan et al. (2006). To extract sediment samples, the Soxhlet extractor was used. Approximately 5 g of sample was added to the extraction chamber, and 120 mL of DCM was added to a round-bottom flask. The mixture was heated for six to 8 hours or cycles for complete extraction, and the extracts were stored in a fume hood before clean-up to prevent loss of volatile extractable compounds Peterson and Freeman (1982).

### Statistical analysis

Data were subjected to descriptive statistics, Students’ t-test, one-way ANOVA. Statistical significance was considered at 0.05 levels of significance. In addition, Principal Component Analysis (PCA) was used to visualize the relationship between metals (fish muscle and sediment) and phthalates (DEHP, DBP, and DEP) in sediment (Adeogun et al., 2015b) for both Lagos and Epe Lagoon (SM II). Prior to the analysis, data transformation procedures, i.e., standardization were applied to ensure that all variables have the same weight in the analysis. The PCA was conducted using Statistica^®^ version 12 software. The output of the analysis provided a visual representation of the relationship between metals, physicochemical properties and phthalate contaminants in the lagoons. All other analysis was achieved using SPSS^®^, and GraphPad Prism^®^ 5.

### Results

#### Biometric assessment

A total of 195 samples of *C. nigrodigitatus* were encountered in this study with Lagos Lagoon accounting for 53.84%, of the total population consisting of 60 males and 45 females while Epe Lagoon accounted for 46.15%, consisting of 45 males and 45 females (Table 1). Males from Lagos Lagoon were significantly longer and heavier than females, while for Epe Lagoon, males were heavier than females. The GSI was significantly higher in females than males across both lagoons (Table 2). Condition factor (CF) for males and females at two locations, Lagos and Epe revealed higher values in males than females at both locations. Additionally, condition factor at Epe was higher for both males and females compared to Lagos Lagoon.

**TABLE 1:** Distribution and abundance of *Chrysichthys nigrodigitatus* from Lagos and Epe lagoons.

	Male	Female	Total
Lagos lagoon	60	45	105
Epe lagoon	45	45	90
Total	105	90	

**TABLE 2:** Total length (TL), body weight (BW) and gonad somatic index (GSI) of *Chrysichthys nigrodigitatus* in Lagos and Epe lagoons.

Location	Sex	TL (cm)	BW (g)	GSI	Condition factor
Lagos Lagoon	Male	28.28 ± 1.97^a^ (13.30–134.00)	160.44 ± 16.00^a^ (21.00–603.00)	0.21 ± 0.12^a^ (0.06–0.90)	0.79 ± 0.25 (0.72–1.47)
	Female	25.03 ± 0.91^a^ (15.8–41.8)	146.88 ± 20.12^a^ (31.00–715.70)	0.47 ± 0.37^a^ (0.10–1.20)	0.77 ± 0.06 (0.66–0.82)
Epe Lagoon	Male	28.12 ± 0.55^a^ (21.50–39.60)	207.02 ± 11.22^b^ (90.00–464.80)	0.11 ± 0.02^a^ (0.01–0.15)	0.90 ± 0.35 (0.75–1.64)
	Female	35.12 ± 6.74^a^ (15.8–41.80)	222.32 ± 12.31^b^ (130.00–580.00)	0.52 ± 2.75^b^ (1.02–9.30)	0.86 ± 0.09 (0.75–0.98)

Note: Values in the table are represented as mean ± standard deviation. Values within parentheses represent the range of the data. Values with different superscripts (a,b) within the same row are significantly different (*p* < 0.05).

#### Gonad pathology and intersex

**FIGURE 2:**
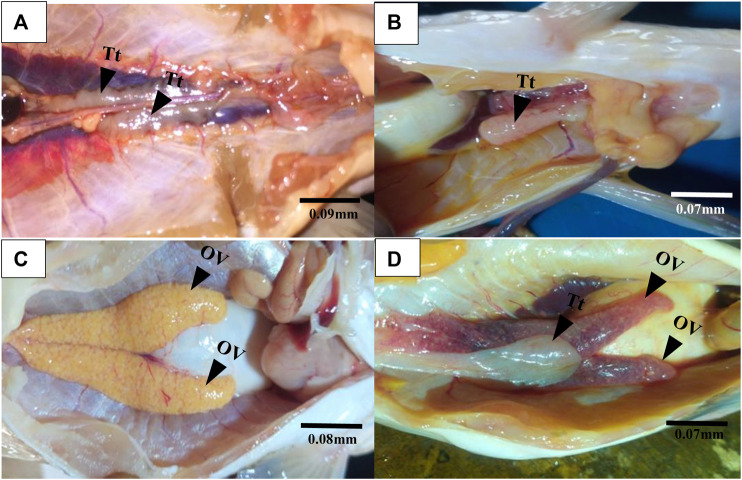
Gross morphological examination of gonads from Lagos and Epe lagoons. **(A)** Normal male *C. nigrodigitatus* from Lagos Lagoon with a pair of testis (Tt). **(B)** male *C. nigrodigitatus* from Lagos Lagoon with one testis (Tt). **(C)** Normal female *C. nigrodigitatus* from Epe Lagoon with a pair of ovaries (OV). **(D)** Intersex female *C. nigrodigitatus* from Epe Lagoon showing a pair of ovaries (OV) alongside a testis (Tt).

**FIGURE 3:**
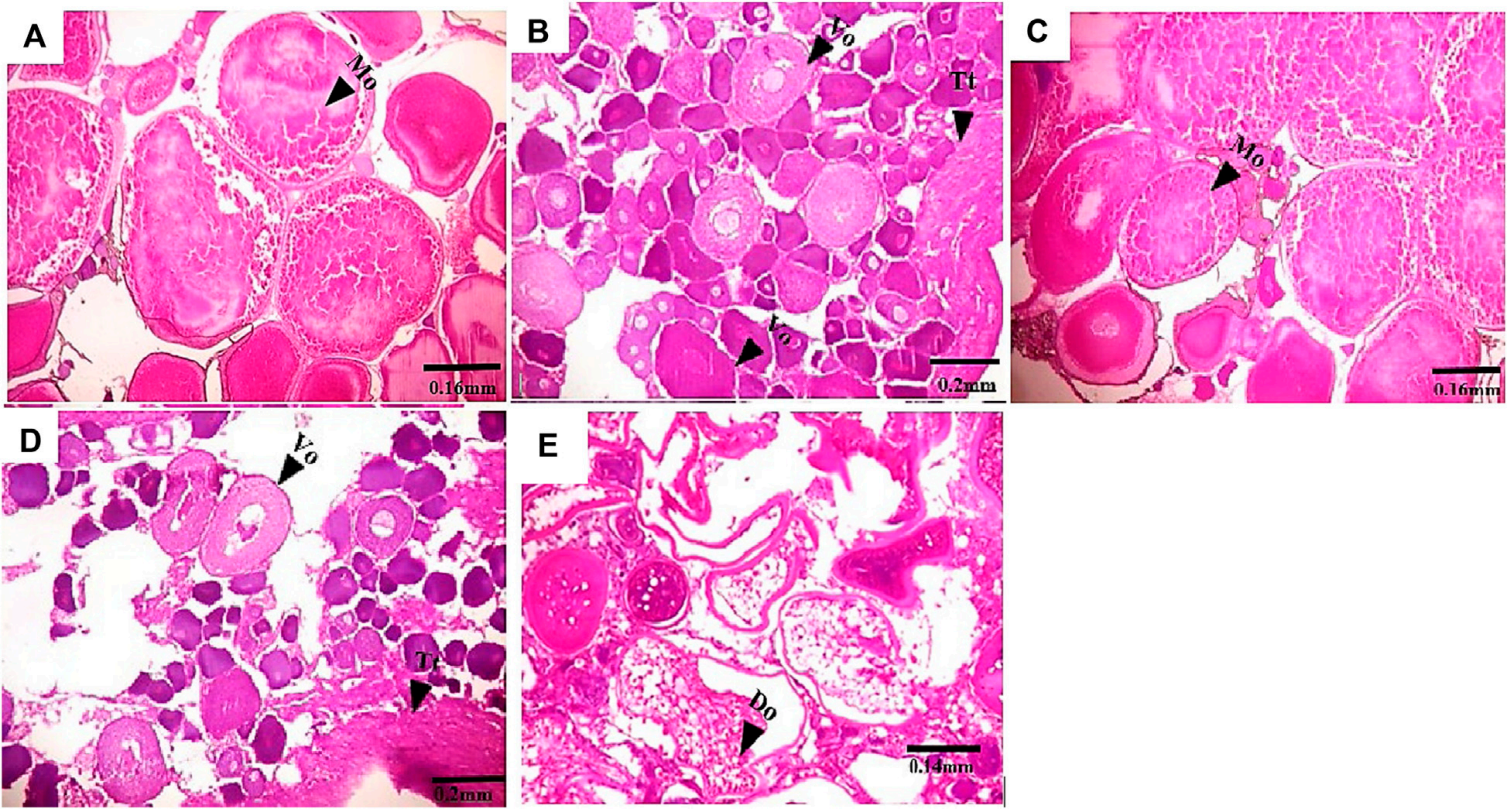
Transverse section of *C. nigrodigitatus* ovaries from Lagos and Epe lagoons. **(A)** Normal ovary from Lagos Lagoon with mature ovary (Mo). **(B)** Intersex female ovary from Lagos Lagoon showing vitellogenic oocyte (Vo) present alongside testicular tissues (Tt). **(C)** Normal ovary from Epe Lagoon showing mature oocyte (Mo). **(D)** Intersex ovary from Epe Lagoon showing Vitellogenic oocyte (Vo) present alongside testicular tissues (Tt). **(E)** Ovary from Epe Lagoon showing distorted ovaries (Do).

**FIGURE 4:**
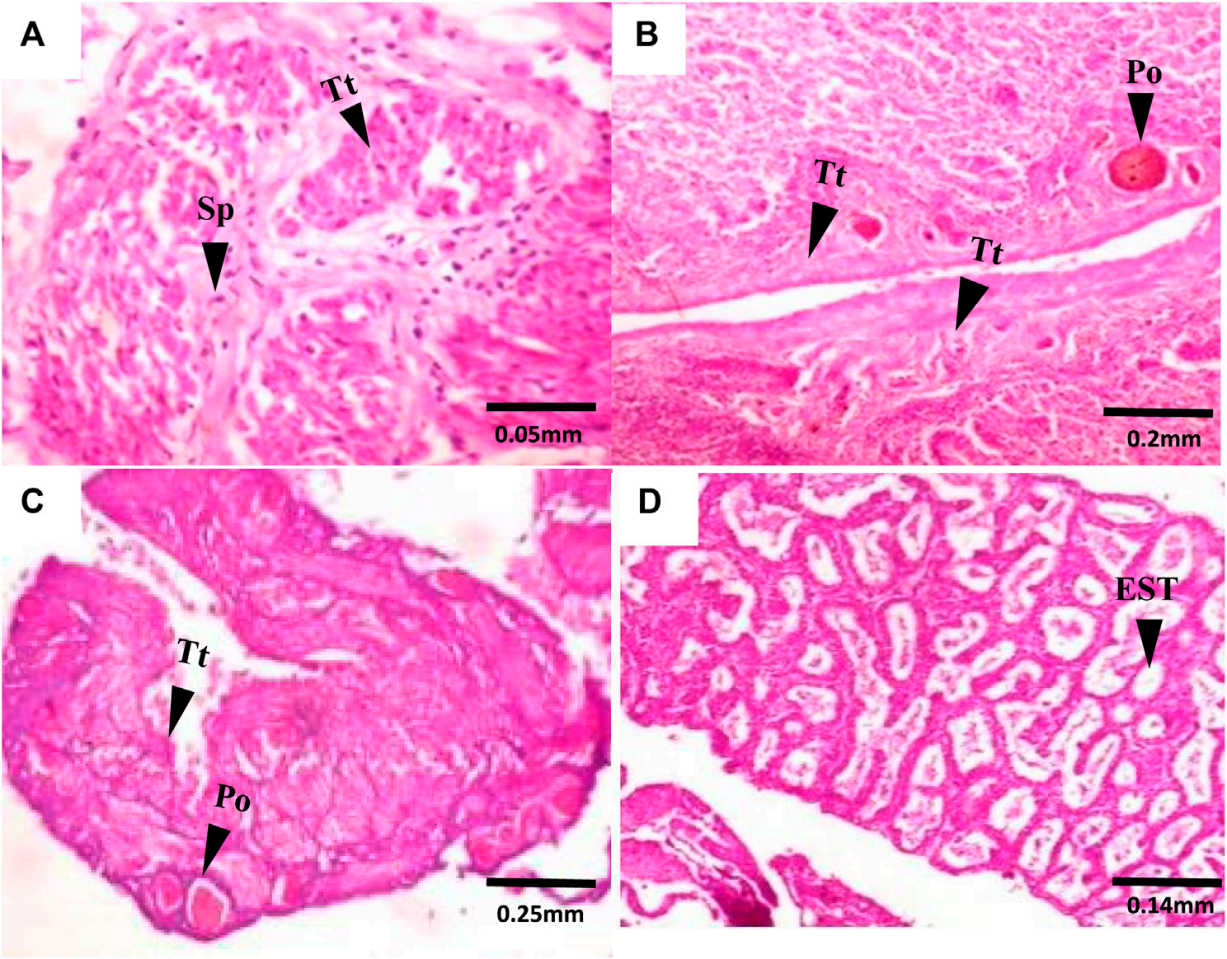
Transverse section of C. nigrodigitatus Testes from Lagos and Epe lagoons: **(A)** Normal testis from Lagos Lagoon showing testicular tissue (Tt) and Spermatid (Sp). **(B)** Intersex testis from Lagos Lagoon showing Primary oocyte (Po) present alongside testicular tissues (Tt). **(C)** Intersex testis from Epe Lagoon showing Primary oocyte (Po) present alongside testicular tissues (Tt). **(D)** Testis from Epe Lagoon showing empty Seminiferous tubule (EST).

Gross morphological examination of *C. nigrodigitatus* gonads showed the occurrence of gonad alteration in samples from both Lagos and Epe lagoons (Figure 2). A male fish gonad with one testis was observed in samples from Lagos Lagoon while an Intersex female showing a pair of ovaries alongside a testis was observed in Epe Lagoon samples. Pathological examination of gonads showed the occurrence of oocytes alongside testicular tissues in both female (Figure 3) and male gonads (Figure 4) of *C. nigrodigitatus* from Lagos and Epe lagoons respectively. Intersex was observed in 3.81% of Lagos (males: 50%; females: 50%) and 3.33% Epe (males: 33.3%; females 66.7%) lagoons fish population respectively. Other gonadal alterations ranged from empty seminiferous tubules to dead spermatids in males and distorted ovaries in females in these two lagoon ecosystems (Figure 4).

#### Serum hormone and vtg protein levels

**FIGURE 5:**
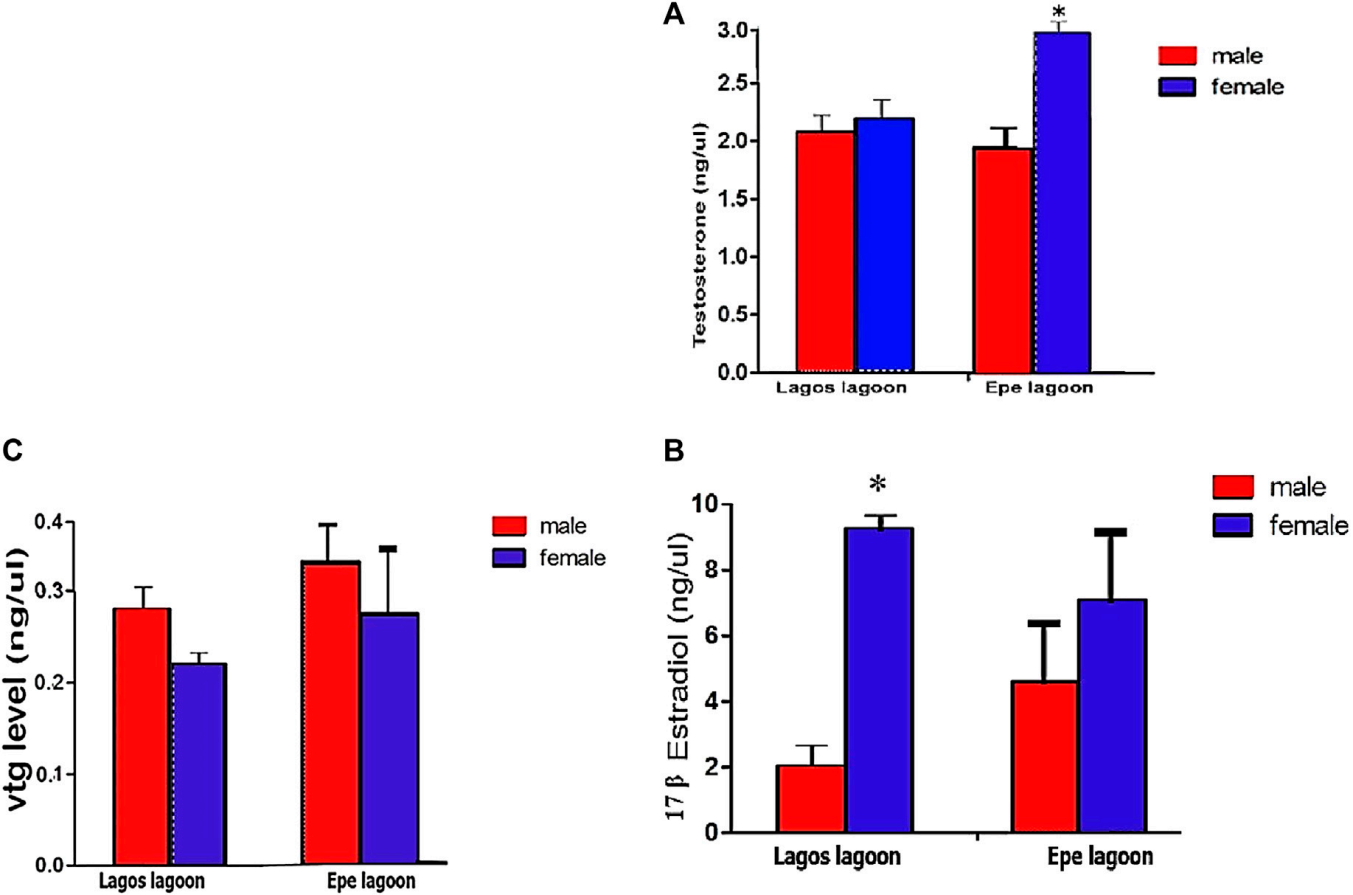
Concentrations of **(A)** Testosterone (ng/μL) **(B)** 17-βestradiol level (ng/μL) and **(C)** Plasma Vitellogenin levels in *Chrysichthys nigrodigitatus* from Lagos and Epe lagoons analyzed using Students’s t-test. Error bars represent standard error of mean (SEM). The level of significance was set at *p* < 0.05. * significant difference between sexes.

Serum hormone levels showed sex-related variations and an indication of hormonal disruption in *C. nigrodigitatus* from Lagos and Epe lagoons with females having a higher testosterone level than males in both lagoons (Figure 5). Testosterone (ngL^−1^) levels in female fish (2.20 ± 0.36) were slightly higher than males (2.16 ± 0.31) in *C. nigrodigitatus* from Lagos Lagoon. In Epe Lagoon, however, the serum testosterone levels were significantly higher in females (3.00 ± 0.71) than male (1.94 ± 0.48) fish (Figure 5A). For 17β-estradiol (E2: ngL^−1^), females showed higher levels in both lagoons (Lagos: female: 9.24 ± 0.82; male: 2.02 ± 0.57; Epe: female: 7.07 ± 2.26; male: 4.60 ± 1.89) with Lagos Lagoon *C. nigrodigitatus* having a higher E2 level than fish from Epe Lagoon (Figure 5B). Vitellogenin protein (ngL^−1^) levels also showed sex-related variations in *C. nigrodigitatus* from Lagos and Epe lagoons with the male having a higher Vtg level than females in both lagoons. This is also an indication of hormonal disruption given that vitellogenesis is a female-specific process (Figure 5C). Vitellogenin levels in males (0.28 ± 0.08) were significantly higher than in females (0.23 ± 0.02) in *C. nigrodigitatus* from Lagos Lagoon while in Epe Lagoon the Vtg levels were also higher in males (0.31 ± 0.03) than in female (0.28 ± 0.0.5) fish (Figure 5C).

#### Physicochemical properties

The mean and standard deviation of various water quality parameters across the two sites, Lagos and Epe Lagoons, are presented in Supplementary Material SII. In Lagos Lagoon, the pH value was 7.35 ± 0.01, indicating slightly alkaline water. The mean dissolved oxygen (DO) value was 10.33 ± 0.22, indicating well-oxygenated water. The conductivity value was 37.73 ± 0.1, relatively low, while total dissolved solids (TDS) was 32.83 ± 1.80, indicating low mineralization levels in the water. In contrast, Epe Lagoon had a lower pH value of 7.1 ± 0.17, indicating a wider variation in pH. The DO mean value was 3.97 ± 0.1, indicating relatively low levels of oxygen in the water. Conductivity values were much higher than that of Lagos Lagoon, with a value of 1,060.33 ± 1.80, indicating more mineralization in the water. The mean value of TDS was 549 ± 2.29, which was also higher than that of Lagos Lagoon (Supplementary Material SII).

#### Metals in sediment and muscle of *Chrysichthys nigrodigitatus* from Lagos and Epe lagoons

Table 3 shows the level of five toxicological relevant metals (Cr, Cd, Pb, As, and Hg: mg/g) measured in sediment from Lagos and Epe lagoons. Chromium levels were higher in Lagos Lagoon (0.21 ± 0.00) than in Epe Lagoon (0.19 ± 0.00). The levels of Cadmium were higher in Epe Lagoon (0.02 ± 0.00) than in Lagos Lagoon (0.01 ± 0.00). Lead levels were higher in Lagos Lagoon (0.42 ± 0.00) than in Epe Lagoon (0.39 ± 0.00). Arsenic levels were higher in Epe Lagoon (0.21 ± 0.02) than in Lagos Lagoon (0.14 ± 0.01). Mercury levels were also higher in Epe Lagoon (0.08 ± 0.01) than in Lagos Lagoon (0.04 ± 0.01).

**TABLE 3:** Concentration of heavy metals in sediment from Lagos and Epe lagoons.

Location	Cr (μg/g)	Cd (μg/g)	Pb (μg/g)	As (μg/g)	Hg (μg/g)
Lagos	0.21 ± 0.00	0.01 ± 0.00	0.42 ± 0.00	0.14 ± 0.01	0.04 ± 0.01
Epe	0.19 ± 0.00	0.02 ± 0.00	0.39 ± 0.00	0.21 ± 0.02	N/A
WHO, 1993	N/A	0.6	N/A	N/A	0.08 ± 0.01

Note: Different letters denote significant difference (*p* < 0.05) across different standards (μg/g). N/A = not available.

Tissue concentrations of five toxicological-relevant metals (Cr, Cd, Pb, As, and Hg) and arsenic measured in male and female *Chrysichthys nigrodigitatus* from Lagos and Epe lagoons were generally higher in females compared to males for both Lagos and Epe lagoons (Table 4). Metal concentrations were also above the limits specified for food by Food and Agricultural Organization and World Health Organization (Joint FAO/WHO, 2011). In Lagos Lagoon females had higher Chromium levels (0.10 ± 0.12 μg/g) than males (0.02 ± 0.00 μg/g). Cadmium levels were higher in females (0.01 ± 0.00 μg/g) than in males (0.00 ± 0.00 μg/g). Lead levels were higher in males (0.35 ± 0.00 μg/g) than in females (0.17 ± 0.00 μg/g). Arsenic levels were higher in females (0.02 ± 0.00 μg/g) than in males (0.01 ± 0.01 μg/g). Mercury levels were also higher in females (0.02 ± 0.01 μg/g) than in males (0.01 ± 0.00 μg/g). In Epe Lagoon Chromium levels were higher in females (0.23 ± 0.00 μg/g) than in males (0.18 ± 0.00 μg/g). Cadmium levels were higher in females (0.01 ± 0.00 μg/g) than in males (0.00 ± 0.00 μg/g). Lead levels were also higher in females (0.44 ± 0.00 μg/g) than males (0.35 ± 0.00 μg/g) and females had higher Arsenic levels (0.03 ± 0.00 μg/g) than males (0.02 ± 0.00 μg/g). Mercury levels (0.02 ± 0.00 μg/g) were similar in both male and female fish from Epe Lagoon.

**TABLE 4:** Concentration of heavy metals in the muscle of *Chrysichthys nigrodigitatus* from Lagos and Epe lagoons.

	Chromium (Cr)	Cadmium (Cd)	Lead (Pb)	Arsenic (As)	Mercury (Hg)
Lagos M	0.02 ± 0.00^a^	0.01 ± 0.00^a^	0.35 ± 0.00^a^	0.01 ± 0.01^a^	0.01 ± 0.00^a^
Lagos F	0.10 ± 0.12^a^	0.00 ± 0.00^a^	0.17 ± 0.00^b^	0.02 ± 0.01^a^	0.02 ± 0.01^a^
Epe M	0.18 ± 0.00^a^	0.00 ± 0.00^a^	0.35 ± 0.00^a^	0.02 ± 0.01^a^	0.02 ± 0.00^a^
Epe F	0.23 ± 0.00^a^	0.01 ± 0.00^a^	0.44 ± 0.00^a^	0.03 ± 0.00^a^	0.02 ± 0.00^a^
Joint FAO/WHO 2011	0.005	0.03	0.5	0.02	0.005

Different letters denote significant differences (*p* < 0.05) across lagoons. M = male; F = Female. Joint FAO/WHO, standards (μg/g).

### Multivariate analysis

**FIGURE 6:**
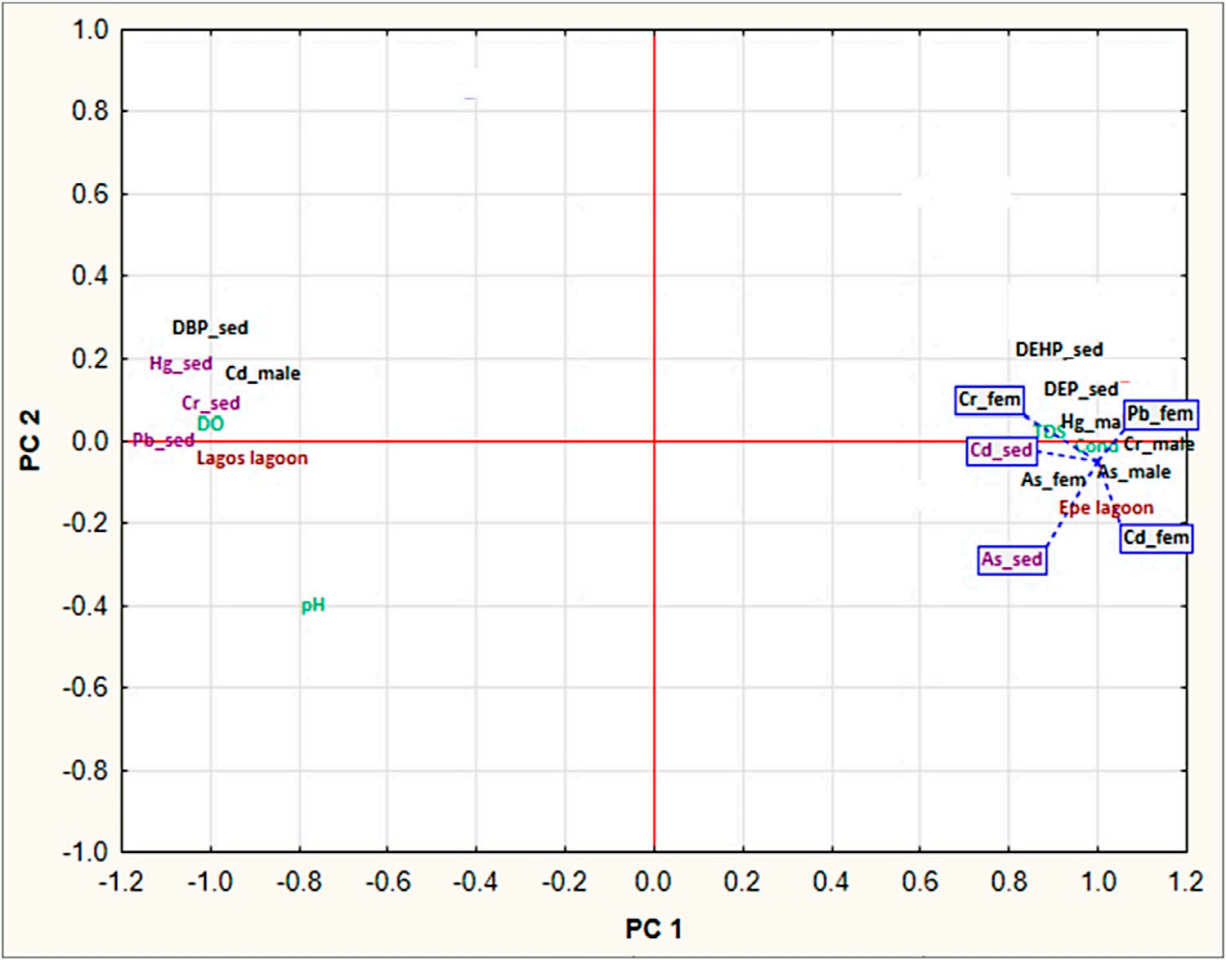
Principal component analysis (PCA) of metal and phthalate contaminants in fish muscle and sediment.

The PCA analysis performed in this study generated five principal components (PCs) that captured most of the variance in the dataset. Among these PCs, PC1 accounted for 89.75% of the total variation in relationship between contaminant trends in fish and sediment of Lagos and Epe lagoon environments. Analysis of the variable loadings in PC1 revealed strong positive associations with two sediment phthalate levels (DEP and DEHP), metals (Hg male, As male, As female, Cd female, Cr male, Cr female, Pb female) in fish muscle and physicochemical parameters (Conductivity and TDS) in Epe lagoon while one phthalate (DBP) and metals in sediment (Cr, Pb, Hg) and muscle metal levels (Cd male) showed strong positive associations with Lagos lagoon environment. The positive values of these loadings suggest that higher concentrations of these compounds are associated with Epe lagoon (Figure 6) (SM 4). This implies that the use of phthalate-containing products may be more prevalent in Epe lagoon, leading to higher levels of phthalate contamination in the area.

While the Lagos lagoon was associated with high loadings of pH, conductivity and TDS were associated with high phthalate ester concentrations in Epe lagoon. The high loading of DPB sediment as the only phthalate with marked trends at Lagos lagoon (Makoko), while DEHP and DEP in sediments associated with Epe lagoon is notable. Overall, PC1 primarily captured variability in the concentration of phthalate esters and pH across the different samples (Figure 6). Furthermore, Lagos lagoon shows sex-specific trends in metal uptake where associations with high positive loadings for Cd in male *Chrysichthys* fish was observed. Epe lagoon on the other hand did not show sex-specific trends as this environment was associated with high loadings of both metals and phthalates in both sexes.

## Discussion

### Gross gonadal anomalies

Gross gonadal examination of *C. nigrodigitatus* from Lagos Lagoon revealed occurrences of gonadal anomalies including of intersex, enlarged gonad, and gonad with only one testis. Epe population on the other hand showed a singular occurrence of a female fish gonad consisting of a pair of ovaries alongside a testis. Similar phenomena have been documented in gonad of fish exposed to estrogenic wastewater effluents indicating exposure to xenobiotics (Woodling et al., 2006; Vajda et al., 2008). However, such co-occurrence of gonads in non-hermaphroditic species are suggestive of intersex due to endocrine disruptive effects (Adeogun et al., 2019).

Furthermore, lesions in gonadal tissue including empty seminiferous tubules, degenerating spermatids and distorted ovaries observed in this study may be linked to exposure of fish to estrogenic and testicular growth inhibition and degeneration agents (Kunz et al., 2006; Rey Vázquez et al., 2009). Empty seminiferous tubules may eventually affect the ability of male fish to produce spermatocytes which ultimately disrupts spermatogenesis. Fish with abnormal seminiferous tubules has been reported to not contain spermatocytes at any reproductive stage (Ünal et al., 2007). Furthermore, distorted ovaries and other ovarian abnormalities have been attributed to reproductive failure in fish (Nash et al., 2004; Abdel-Moneim et al., 2015). Fewer spermatogenic cysts in testis and much fewer mature follicles in ovaries of fish exposed to UV filter 3-benzylidene camphor have been demonstrated to have a significant effect on gonadal development, fertility and reproduction of fish with potential consequences at the population level (Kunz et al., 2006). Exposure of African catfish, *Clarias gariepinus* to EDCs in the wild has resulted in vacuolization of the seminiferous tubules, empty and disintegrated seminiferous tubules, degeneration of germ cells, and hypertrophy of Sertoli cells (Bhaisare et al., 2022).

Although the marginally higher intersex occurrence in Lagos Lagoon *C. nigrodigitatus* (3.81%) compared to Epe Lagoon (3.33%) confirms reports of endocrine disruption events among Lagos and Epe Lagoons fish populations (Adeogun et al., 2015b), it also suggest that the magnitude of effects due to pollution are similar for both lagoons. Intersex has also been reported in similar studies in which fish were exposed to EDCs from their natural habitat. Harris et al. (2011), reported the occurrence of intersex in Adult roach (*Rutilus rutilus*) from wild populations living in effluent-contaminated rivers in the United Kingdom. Adeogun et al. (2016b) reported 33% and 34% intersex and alterations in reproductive development of tilapia species from two municipal domestic water supply Lakes (Eleyele and Awba) in Southwestern Nigeria. Furthermore, studies on development in *Sarotherodon melanotheron* fish from Lagos lagoon showed a 27.4% prevalence of intersex, with severe evidence feminization of male fish (Adeogun et al., 2019).

Intersex patterns observed in this study is an indication that females had a higher occurrence when compared with males suggesting that females may be more susceptible to EDCs than males. This is consistent with the reports on benthic and pelagic fish from the Owan River in south-south Nigeria which recorded a higher incidence of intersex and gonadal anomalies in female fish (42%) compared to males (12%) (Chukwuka et al., 2019).

#### Biochemical evidence of endocrine disruption

The significantly higher 17β-estradiol level recorded in male fish from Epe Lagoon compared to male fish from Lagos lagoon indicates higher likelihood for feminization of male fish. On the other hand, the lower average levels of 17β-estradiol in Epe female fish compared to Lagos lagoon fish indicates masculinization of females. This possibility is corroborated by the higher male vtg levels compared to female and significantly lower testosterone levels in males compared to females. The relationship between elevated testosterone levels in female fish and development of intersex condition have been linked with xenoestrogenic effects (Jobling et al., 2003). Feminization in male fish has been also been associated with the presence of estrogenic substances in water (Kidd et al., 2007). Xenoestrogens exert its effect on sex differentiation by altering the expression level of steroidal receptors and steroidogenic enzymes.in addition to influencing steroidogenesis and steroid receptor expression. They also act directly on sex-determining genes and thus influence sex differentiation in gonochoristic species. Furthermore, female fish encountered in this study generally showed higher testosterone levels than 17β-estradiol with potential negative consequences on the quality of oocytes produced. Bugel et al. (2011) correlated decreased 17β-estradiol levels with inhibition of oocyte development and decreased sensitivity of the Vtg pathway.

While the testosterone and vtg levels in male and female fish from the Lagos lagoon showed abnormalities with no significant difference between them, the levels of 17β-estradiol were significantly higher in females than males, indicating that the mechanisms of endocrine disruption in fish populations in the Lagos and Epe lagoons may differ. The changes in 17β-estradiol levels are typically associated with endocrine disruption through estrogenic pathways (E Haut, 2005). However, the observed patterns in Lagos lagoon suggest the possibility of a different steroidogenic pathway, likely influenced by a distinct type of xenoestrogenic exposure. As xenoestrogens exhibit varying structural complexity and produce numerous metabolites or biodegradation products in the environment, they are capable of displaying a range of mechanisms of action (Kerdivel et al., 2013).

#### Multivariate relationships

The implications of the findings from the PCA analysis in this study are significant for ecological effects. The higher concentration of phthalate esters and metals in sediment at Epe lagoon may imply greater bioavailability, thus portending risks of reproductive failure, population declines and disruptions in aquatic food web. pH had a relatively high loading associated with Lagos lagoon in PC1 also has important ecological implications on the bioavailability and toxicity of metals and other contaminants in the aquatic environment. The higher loading for pH implies greater alkalinity at the Lagos lagoon site, which may explain why the Lagos lagoon site was predominantly associated with metals in sediments and DBP in sediment. Lower pH values can increase the solubility and toxicity of metals, while higher pH values can decrease their bioavailability and toxicity (Peng et al., 2009). In particular, pH dictates metal speciation, influencing their reactivity and toxicity. In acidic conditions, protons compete for ligand binding, yielding more toxic free metal ions, while alkaline conditions promote less reactive metal-ligand complexes or precipitates, reducing toxicity (Namieśnik and Rabajczyk, 2010). Furthermore, the association of high phthalate ester concentrations with conductivity and TDS suggests that these physicochemical parameters may play a role in the transport and fate of phthalate esters in the aquatic environment at the Epe site (Zheng et al., 2014). The elevated ion content, indicated by increased conductivity, has significant implications for the fate of phthalate esters by potentially enhancing their mobility and dispersion in the aquatic environment through the formation of ion-pair complexes, thereby affecting their solubility and facilitating movement through water (Dueñas-Moreno et al., 2022; Zhu et al., 2022). This heightened mobility and dispersion of phthalate esters in the aquatic environment, facilitated by increased ion content and ion-pair complex formation, may elevate the risk of organism uptake, influencing their exposure to these contaminants (Sardiña et al., 2019).

Since phthalates are known to be endocrine disruptors that can affect the reproductive and developmental processes of aquatic organisms, leading to population declines and ecosystem instability, species at Epe may be at greater risks of reproductive toxicity that the Lagos lagoon site. On the other hand, the high loadings of metals in sediment samples from Lagos lagoon, particularly Hg_sed, Pb_sed, Cr_sed, and DPB_sed, suggest that the contamination of Makoko area of the Lagos lagoon for these metals is a site-specific feature. This further implies that exposures of resident fish fauna to this phthalate and metal species could be chronic, since sediment repository can ensure sustained exposures over time. These metals could eventually accumulate in the tissues of local fish populations, leading to toxicity and biomagnification in higher trophic levels.

To further understand the ecotoxicological implications of the findings, it is worth noting that the sex-specific trends observed in metal uptake in Lagos lagoon may be attributed to the ecological features or habitat terrain of the site at Makoko. The high positive loadings for Cd in male Chrysichthys fish suggest that male fish in Lagos lagoon may exhibit sex-specific ecological behavior, which can result in sex-specific contaminant uptake and toxicity (Jerome et al., 2017b). This is an important finding that underscores the need to consider sex-specific responses in ecotoxicological studies. By contrast, Epe lagoon fish did not show any sex-specific trends in metal uptake but was associated with high loadings of both metals and phthalates in both sexes. Overall, the PCA analysis highlights the complex relationships between environmental variables and contaminants in the Lagos and Epe lagoon systems, providing insights into the sources and pathways of contamination in these aquatic ecosystems. The association of conductivity and TDS with high phthalate ester concentrations in Epe lagoon suggests that these physicochemical parameters may be influencing the transport and fate of phthalates in the environment. High conductivity and TDS can increase the solubility and mobility of contaminants, leading to increased exposure and potential harm to aquatic organisms.

## Conclusion

In this study we have demonstrated that pollution of Lagos and Epe lagoons may have negative effects on *Chrysichthys nigrodigitatus*, with phthalates and heavy metals identified as significant pollutants. The discharge of industrial and anthropogenic effluent containing these contaminants results in hormonal imbalances in male and female fish, as shown by disrupted steroid hormone levels and Vtg detection in males. Both male and female fish also experience gonad alterations. Therefore, this study provides insights into the sources and pathways of contamination in these tropical aquatic ecosystems, which may explain the site-specific occurrences of gonadal alterations in male and female fish. Targeted management and remediation efforts can be informed by identifying specific contaminants and their temporal and spatial trends.

## Data Availability

The original contributions presented in the study are included in the article/Supplementary Material, further inquiries can be directed to the corresponding author.
