# Supplementary material for: Gonad pathology, sex hormone modulation and vitellogenin expression in Chrysichthys nigrodigitatus from Lagos and Epe lagoons within the southern-lagoon system, Nigeria
Source: Front Toxicol. 2024 Feb 6;6:1336916. doi: 10.3389/ftox.2024.1336916 (PMC10878419; doi:10.3389/ftox.2024.1336916)
Supplement: Supplementary file 1 [file Table1.docx]

**Supplementary information I**

**Supplementary material (SM) I: ELISA Standard curve for Optical density (OD) and concentration**

**
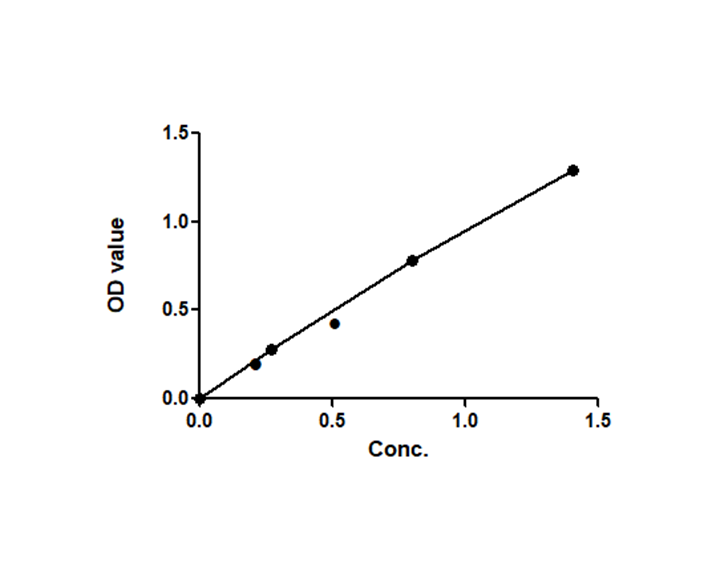
**

**Supplementary information II**

**Physicochemical properties**

| Site |  | Mean | Std. Deviation |
| --- | --- | --- | --- |
| Lagos lagoon | **pH** | 7.35 | 0.01 |
|  | **DO** | 10.33 | 0.22 |
|  | **Cond** | 37.73 | 0.10 |
|  | **TDS** | 32.83 | 0.20 |
| Epe lagoon | **pH** | 7.10 | 0.17 |
|  | **DO** | 3.97 | 0.10 |
|  | **Cond** | 1060.33 | 1.80 |
|  | **TDS** | 549.00 | 2.29 |


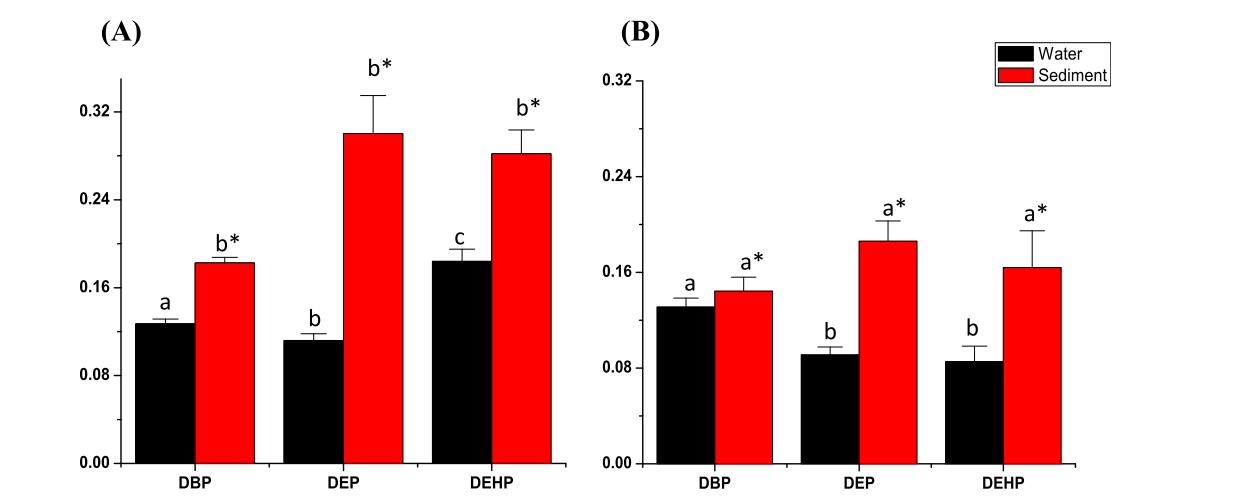


**Adeogun et al., 2015b: Concentration of phthalate esters (PEs) in water (mg/L) and sediment (mg/kg) from the Epe (A) and Lagos (B) lagoons, Nigeria. Values represent mean ± standard deviation (SD; n ¼ 3). Different letters indicate significant difference (p < 0.05) between the 3 classes of PEs (DBP, DEP, DEHP) analyzed using one-way ANOVA, while asterisk (*) indicate significant difference between the concentrations of individual PE in water and sediment by paired student t-test, performed using Origin 8 software (OriginLab, USA). The level of significance was set at p < 0.05.**

**Supplementary material III**

**principal component scores**

|  | **PC 1** | **PC 2** | **PC 3** | **PC 4** | **PC 5** |
| --- | --- | --- | --- | --- | --- |
| DEHP_sed | 0.941 | 0.136 | -0.230 | -0.054 | 0.201 |
| DEP_sed | 0.997 | 0.055 | 0.047 | 0.015 | 0.025 |
| DBP_sed | -0.979 | 0.197 | 0.025 | 0.042 | -0.022 |
| pH | -0.768 | -0.433 | -0.424 | 0.216 | 0.002 |
| DO | -0.999 | 0.004 | 0.039 | -0.031 | 0.010 |
| Cond | 0.998 | -0.048 | -0.019 | 0.042 | -0.014 |
| TDS | 0.997 | -0.051 | -0.021 | 0.042 | -0.019 |
| Cr_sed | -0.998 | 0.047 | 0.018 | -0.041 | 0.015 |
| Cd_sed | 0.998 | -0.047 | -0.018 | 0.041 | -0.015 |
| Pb_sed | -0.998 | 0.047 | 0.018 | -0.041 | 0.015 |
| As_sed | 0.998 | -0.047 | -0.018 | 0.041 | -0.015 |
| Hg_sed | -0.998 | 0.047 | 0.018 | -0.041 | 0.015 |
| Cr_male | 0.998 | -0.047 | -0.018 | 0.041 | -0.015 |
| Cd_male | -0.998 | 0.047 | 0.018 | -0.041 | 0.015 |
| As_male | 0.998 | -0.047 | -0.018 | 0.041 | -0.015 |
| Hg_male | 0.998 | -0.047 | -0.018 | 0.041 | -0.015 |
| Cr_fem | 0.998 | -0.047 | -0.018 | 0.041 | -0.015 |
| Cd_fem | 0.998 | -0.047 | -0.018 | 0.041 | -0.015 |
| Pb_fem | 0.998 | -0.047 | -0.018 | 0.041 | -0.015 |
| As_fem | 0.998 | -0.047 | -0.018 | 0.041 | -0.015 |
| Site_Lagos lagoon | -0.998 | 0.047 | 0.018 | -0.041 | 0.015 |
| Site_Epe lagoon | 0.998 | -0.047 | -0.018 | 0.041 | -0.015 |
| **% Total variance** | **89.746** | **5.128** | **3.631** | **1.174** | **0.322** |
| **Cumulative %** | **89.746** | **94.874** | **98.505** | **99.678** | **100.000** |
